# Spread of Makoyoh’sokoi (Wolf Trail): a community led, physical activity-based, holistic wellness program for Indigenous women in Canada

**DOI:** 10.1186/s41043-023-00427-w

**Published:** 2023-08-12

**Authors:** Levi Frehlich, Ashley Amson, Patricia Doyle-Baker, Tia Black, Dawn Boustead, Erin Cameron, Lynden (Lindsay) Crowshoe, Kerry McBrien, Yunqi (Jacob) Ji, Ashlee McGuire, Alicia Oliver, Loretta Tuttauk, Jessica Zhang, Carly Checholik, Sonja Wicklum

**Affiliations:** 1 University of Calgary, AB Calgary, Canada; 2 University of Ottawa, Ottawa, ON Canada; 3 Miskanawah, Calgary, AB Canada; 4 Northern Ontario School of Medicine, Sudbury, ON Canada; 5 Onion Lake Health Board, Onion Lake, SK Canada; 6 University of Alberta, Edmonton, AB Canada

**Keywords:** Education, Health, Indigenous, Physical activity, Wellness, Women

## Abstract

Globally, Indigenous populations have been impacted by colonization. Populations who have endured colonization are at higher risk of developing chronic diseases. Canada’s Truth and Reconciliation Commission emphasizes reducing barriers to participation in physical activity and recommends the creation of culturally relevant and supportive policies and programing. Physical activity is a cornerstone in health promotion and public health to combat chronic diseases; however, in Canada, Indigenous developed physical activity programing is sparse, and those targeting women are non-existent in some regions. Makoyoh'sokoi (The Wolf Trail Program) is an 18-week long, holistic wellness program that was created by and for Indigenous women. Makoyoh'sokoi was developed by communities following extensive consultation and cultural oversight. Makoyoh'sokoi’s core program consists of 12 weeks of weekly physical activity programing and health education, followed by another 6 weeks of weekly health education. Notably, communities have control over the program to modify based on individual needs and challenges. Programs commence and conclude with a ceremony with Elders giving a blessing and opening each other to connection. The goals of Makoyoh'sokoi are to empower women, improve health outcomes, and to implement a sustainable program by training a network of community members in their respective communities to facilitate delivery.

## Introduction

Globally, there is a need to recognize the harms and impacts of colonization because of the impact it has had on all aspects of life, including health, traditional roles, culture, socio-economic conditions, access to services, and equity, among others [1]. Indigenous populations in Canada continue to experience health disparities compared to non-Indigenous Canadians due to colonization, poverty, climate change, loss of language and culture, and disconnection from the land. Compared to the general population, Indigenous people have higher rates of chronic diseases such as type II diabetes, obesity and heart diseases [2]. The forced transition from a traditional way of life has led to poor diets, sedentary lifestyles, and challenges with obesity and other chronic diseases [3].

With colonization also came the introduction of patriarchy, disproportionately impacting women who historically played central roles in society and held valuable knowledge on healthy food systems [4, 5]. A 2019 scoping review of obesity rates among Indigenous Peoples in Canada indicates that obesity rates are commonly higher in women compared to men [6]. An analysis of Statistics Canada's Aboriginal Peoples Survey 2012 found Indigenous women have comparatively worse self-reported health [7]. A community-based and culturally grounded holistic, wellness program for women is one strategy to promote health.

This program aligns with two Calls to Action of Canada’s Truth and Reconciliation Commission (TRC) report, which aim to counteract the harms of colonization and bring awareness and recognition to Indigenous people [8]. The TRC has 94 Calls to Action. Calls to Action #88 and #89 recognize a role for physical activity and sport in reconciliation, emphasizing the need to reduce barriers to Indigenous participation, and to create inclusive and culturally relevant physical activity policies, programs, and initiatives [8]. There is substantial evidence for the general population supporting a multi-pronged approach (physical activity, nutrition, and behavioral components) to weight management for obesity and diabetes prevention and management [9]. However, the health disparities endured by Indigenous women necessitate tailored and culturally responsive approaches to health promotion [10, 11].

A review by Rice et al. [12] of physical activity-based interventions for diabetes prevention and management in Indigenous peoples in Canada identified 13 interventions focused mainly on rural settings and school-aged children. Only three of the 13 interventions led to an increase in physical activity levels and they concluded that there was a need for well-studied interventions, which are culturally responsive in design [12]. Similar reviews of physical activity-based programming for Indigenous people in Australia and New Zealand [13] and the USA [14] also emphasized the importance of cultural relevancy in programming. Moreover, a recent systematic review that summarized physical activity-based wellness interventions for Indigenous women found only one intervention that was specific to pregnant women [15].

Makoyoh’sokoi (Wolf Trail), an 18-week, holistic wellness program for Indigenous women, was developed in the community to fill the gap in programming for adult Indigenous women and to provide evidence for the importance of culturally safe, community-based programs.

## Makoyoh'sokoi: program development and overview

True to the culture of many Indigenous populations, Makoyoh’sokoi is rooted in connection, story, and sharing. After consultation with our communities and advisory group (comprised of a Traditional Knowledge Keeper, a local Elder, Indigenous and non-Indigenous health professionals, and government officials), the spirit of Makoyoh'sokoi (ma-koy-yoh- so-koy) was conveyed by the following vision statement:

We **support** Indigenous women to celebrate health and **make a difference in our world.**

**“Support”** the creation of a culturally safe and collaborative environment that addresses physical, mental (intellectual), spiritual, and emotional well-being is central to the Wolf Trail (Makoyoh’sokoi).

**“Make a Difference”** means women who self-activate and support their family, friends, and community. It can come in many different forms, from the prevention of personal ill health to personal growth into positions that may influence the health of others, or to contributing to a supportive healthy community.

**“In Our World”** reflects our understanding of the interconnectedness of all living creatures and the planet we live on, reflecting Indigenous ways of knowing. We also recognize how rapidly the world is changing and how much more interconnected we have become and will continue to be, recognizing that, when one person’s health changes, it can cascade to impact many others both near and far, and for generations to come. By stating this, we acknowledge how the negative impact of colonization cascaded into intergenerational trauma and severe health inequities for Indigenous peoples.

Makoyoh’sokoi was designed specifically to address the health and wellness needs of Indigenous women. Pilot programming, in partnership with seven communities running ten programs across the Canadian provinces of Alberta and Saskatchewan, demonstrated increased weekly pedometer step counts, increased nutritional awareness (consumption of fruits and vegetables), improved confidence in exercising as a group, and improvements in weight and systolic blood pressure [16]. Most notably, participants endorsed the importance of a program that prioritized cultural safety and acknowledged the important role Indigenous women play in their communities [17]. Given this success, expansion and spread of programming began in 2019 and the program was named Makoyoh’sokoi during a traditional Blackfoot naming ceremony.

Makoyoh’sokoi was initially developed in community and expansion was organic, with new sites requesting the program, each with the opportunity to adapt the program to suit their unique needs. Community Leads, individuals with a key role in health promotion within their communities, contributed to the adaptation of the program and the hiring and support of local Program Facilitators. The Program Facilitators administer all aspects of the program, such as hiring fitness instructors, leading the nutrition education, and performing data collection. Together, the Community Leads and Facilitators report back to the participants and their wider community on the outcomes of the program. Additionally, an Advisory Group comprised of Indigenous and non-Indigenous community members and Indigenous Elders oversees the spirit and vision of the program.

**Fig. 1:**
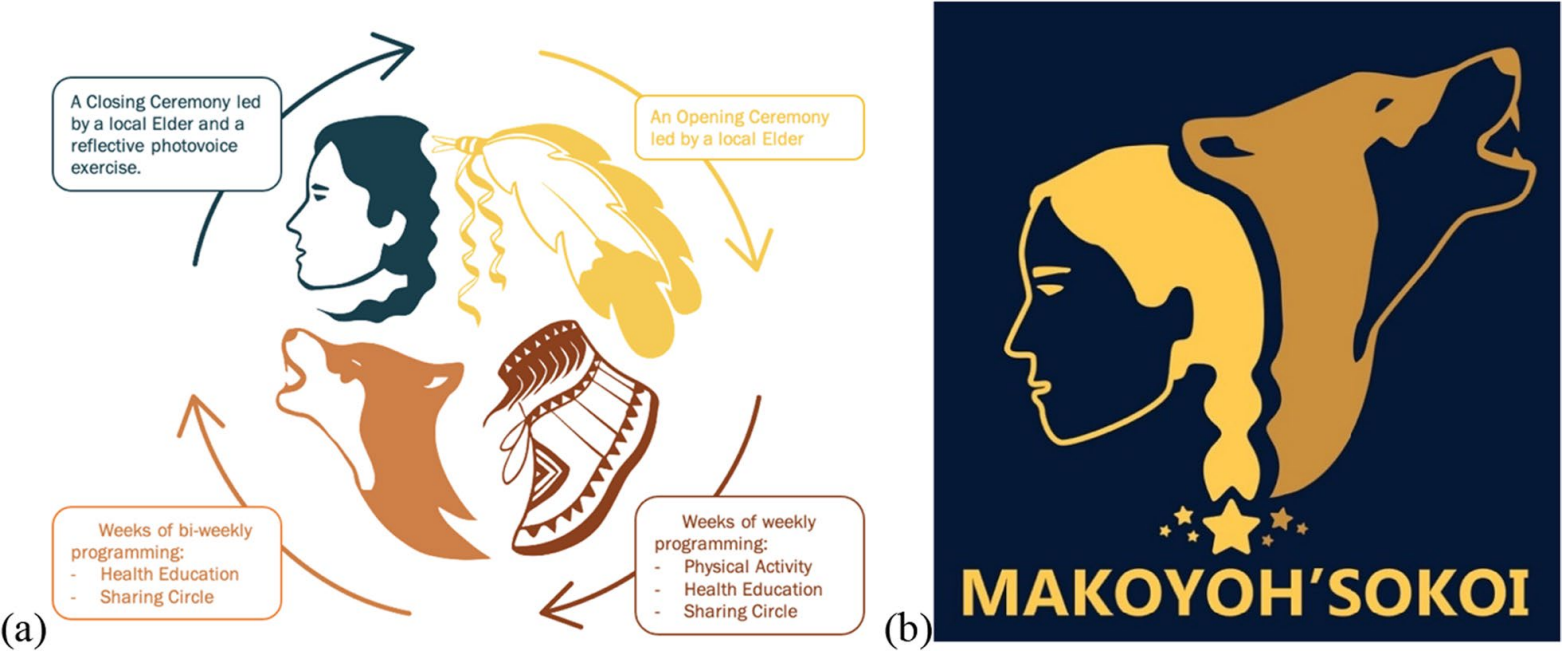
Makoyoh’sokoi program cycle (**a**) and logo (**b**)

Community consultation and cultural oversight was integrated throughout Makoyoh’sokoi development and documentation. Following the naming of Makoyoh’sokoi a call was put out to community members for the development of a logo and media that could be used for program documentation. The result of the community call is shown in Fig. 1, with eagle feathers representing a welcome and Elder teachings, a dancing moccasin representing getting active, the wolf representing connection and community building, and the women representing storytelling and celebration. The main logo for the program is represented by the combination of the wolf and the women along with seven stars representing traditional connections to the sky. In honor of the naming ceremony, every spring there is a celebration that brings together participants, Community Leads, Program Facilitators, and Elders where traditional teachings are shared and an offering to the spirits is made for a successful upcoming program.

With oversight from the advisory group, four manuals were developed for the program including an operational manual, a facilitator manual, and two versions of the *Nourish Health and Well-Being Modules*© (one for Program Facilitators and one for participants). The operational manual includes administrative information such as budgets, and attendance sheets. The Program Facilitator manual provides an overview of the program and information on how to lead a class including how to organize instructors, connect with community leads and Elders, and experimental learning styles. The Nourish manual includes health and well-being modules that the Program Facilitator delivers to participants (Table 1). The Nourish manual was developed with consultation from dieticians, physicians, and Elders. The Nourish manual for Program Facilitators has additional notes and tips for delivery of each module. Moreover, sharing circles are also integrated into every session and commence after a smudge ceremony to create a safe space to connect, offer prayer, and allow time for healing through an Indigenous lens.

**Table 1:** Nourish manual—health and well-being modules

Week	Module	Brief description
	**Initial 12 weeks**	
1	Welcome and elder teachings	This week will welcome participants to the program, get them started with their pedometer, and establish expectations. The Elder will start their group off in a good way, with a spiritual foundation
2	Getting Active!—keeping fun, variety and safety in mind	This week will introduce participants to how to become more active. Getting active is encouraged in many forms such as walking, biking, and sedentary breaks such as setting a timer to get up every hour
3	Understanding your natural healthy self	This week is focused on ‘health intentions’ rather than ‘weight goals’. Health intentions are framed to help participants direct to an action, a direction or a focus that contributes to their health in a positive way such as stretching more often, walking more regularly, or exploring cooking
4	Holistic approach to health—reflection	Makoyoh’sokoi takes a holistic approach to health. This week utilizes the Medicine Wheel to look at the domains of emotional, spiritual, mental and physical health
5	Honoring culture and traditions	This week is about exploring culture and traditions. Facilitators will lead an exploration of local food traditions, discuss why they exist, and if there a spiritual or cultural reason for them
6	Positive eating attitudes	Our ability to regulate food sometimes gets disrupted. Not knowing if there is enough food or when the next meal is, dieting, life stress, and sickness can all mess up food regulation. This week introduces elements of how to build an eating routine that supports healthy eating in the weeks to come
7	Take time to eat	Taking time to eat is very important. And this does not simply refer to the act of eating but it also refers to the planning and preparing. This week focuses on two principles: (1) making time for eating (including planning and prep) and (2) listening to your body and nourishing it when it is needed
8	Putting together a meal	This week focuses on putting together a balanced meal. We do not recommend a specific diet for participants. The principle is to identify some dietary habits that are less healthy and try to move away from these. Also, we aim to identify some key dietary habits that the participant may not be doing now and need to try to incorporate
9	Fiber and grains	This week we will focus on carbohydrates in food. First some general comments and then some more specific information about grains and fiber, breads, and cereals. We also introduce the importance of fiber
10	Vegetables and fruits	This week we introduce vegetables and fruits and touch on traditional fruits. We discuss supplying our bodies with important vitamins, minerals, fiber, energy and an abundance of other phyto (plant) chemicals. We talk about options of obtaining these foods (grow them, buy them fresh, canned or frozen) and how with present day methods of processing they remain full of nutrients in all these forms
11	Protein foods	This week discusses how the body needs protein foods to build and maintain muscle, heal tissue, and for a healthy immune system. Some illnesses and treatments can affect your appetite that may lead to eating less and not meeting your protein needs. Legumes, nuts and seeds, traditional meats and seafood are touched on
12	Beverages and the 1⁄2 way celebration!	During this week participants examine the beverages they consume. Sources of hidden calories and sugar such as juice, specialty coffee, and liquor are discussed. Participants also look back at a previous exercise ‘My Food Journal’ and see if their liquid consumption has it changed
	**Follow-up 6 weeks**	
13	Food skills—menu planning	This week discusses the importance of menu planning. We touch on benefits of planning ahead of time such as allowing others in your home to get engaged in the process, helping to organize shopping, and to reducing waste. A meal plan guide that includes at least 3 breakfasts, lunches, and dinners is included
14	Food skills—grocery shopping and the influence of medications on health	This week gives some tips when going shopping (e.g., don’t shop when you are hungry or in a hurry and go around the outside of the shopping store, this is usually where the fresh vegetables and fruits are, where dairy products and meats are, and you are avoiding a lot of the processed foods on the inside aisles. A health care provider also gives a talk on the importance of medications and how some medications may cause side effects (e.g., weight gain)
15	Relationship with our body and weight	This week starts by looking back at several reflective exercises, looks at all the elements that may influence health and ability to eat well or be active, and at previous attempts at improving health. Using reflective exercises to monitor all four quadrants of your health, health is discussed as more than just weight
16	Self-care for our body	This week provides examples on how to take care of your body that is outside of the program including—physical activity, managing pain, taking time for the things we love that bring us joy, taking time for ourselves, dressing to feel good and for comfort, eating well, and sleeping well
17	When life takes us away from eating well and special social, cultural or mental health guest	During this week we talk about how stressors, transitions and changes in life can cause us to fall out of a healthy routine or change it and how we can reset our health intentions when this happens. We also recommend that this week include a special guest to discuss the mental, emotional, or spiritual sides of holistic health
18	Final day celebration!	The final day is crafted and modified for each community. This day utilizes what the facilitators learned about the participants throughout the previous 18 weeks and ends in a Medicine Wheel Reflective Exercise

The Makoyoh'sokoi program was developed for adult Indigenous women, defined as gender female, and may include cis-female, binary, trans-females, queer and two-spirited individuals. During development, it was noted that participating organizations were very diverse, spanning two provinces (Alberta and Saskatchewan), including communities on- and off-reserve. Therefore, although there are core documents and measures that are proposed for each community, a key novelty of the program rests in the ability of the communities to modify the program based on their unique needs.

Early program development applied social cognitive theory of behavior change, assuming learning from others and being given opportunity to apply new knowledge in a safe environment would support behavior change [17]. Subsequently, in a qualitative evaluation of three years of programming, a realist lens [18] was applied to better understand what contextual elements were supporting effectiveness [19]. Applying realist theory, pilot programming resulted in support for self-actualization through collective effort, improvement in personal wellness, and social and cultural support [19].

Participants are exposed to a variety of physical activity through the course of the program, in part so they can sample and learn what types of activity interests them and gain confidence in their ability to participate. This is done in a safe and culturally relevant environment. They are instructed by individuals that understand their unique needs and social determinants of health. In-between sessions, participants use a step counter and encourage each other (via social media), as well are encouraged by the Facilitator, to improve upon their daily steps.

Moreover, participants complete health education modules that expose them to health information, a substantial portion of which relate to healthy nutrition and disease prevention, and on occasion to local healthcare providers. Again, supported by the safety of the environment, the group can explore their barriers and facilitators to change openly. Specific healthcare needs can then be indicated, and appropriate resources identified. The goal being to increase activation and the ability to manage one’s own health. It is important to note that childhood trauma, addictions, and mental illness all greatly impact an individual’s ability to make positive behavior changes and through pre-program surveys and engagement by Program Facilitators, individuals with more severe, specific needs may be identified and appropriate referrals made. In each community, the Community Organizational Leads will support the Facilitators to establish a comprehensive resource list of healthcare professionals. Through exposure to local Indigenous Knowledge Keepers and Elders, along with cultural and spiritual elements, participants will gain knowledge of how to access activities to further support their wellness journey.

The Makoyoh'sokoi program shows promise as a community-based model for supporting Indigenous women to improve their health. It is grounded in community; increases self-efficacy through opportunity to learn, practice, and share in manageable increments; and increases health literacy through program contents and coordinating healthcare knowledge exchange with other healthcare providers from the community. This is all accomplished in a safe and supportive environment that is therapeutic, enhances connectedness, and fosters collaboration. Lastly, by connecting to aspects of Indigenous culture, participants can be affirmed in their identities and learn more about how connecting to culture may be protective of their health through restored sense of self-worth, sharing, and community support [19].

Each Makoyoh'sokoi program is 18 weeks long divided into two blocks, the first of which is intense programming followed by a less intense follow-up phase. Each program aims to recruit 20–25 participants. The initial 12 weeks consist of (1) a pre-program information and orientation session, and data collection; (2) an opening ceremony with a local Elder; (3) core weekly programming (45–60 min exercise class, 15–20 min nutrition and wellness education***,*** 15–30 min sharing circle); (4) an optional urban pole walking session; (5) introduction to local healthcare providers as identified as relevant for the group (presented during health education component); (6) ongoing online support via social media (moderated by the Program Facilitator with weekly contact mid-way between live sessions); and (7) daily pedometer tracking to provide immediate motivational feedback to participants. The 6-week follow-up phase includes: (1) core weekly programming (15–30 min nutrition and wellness education and 30–45 min sharing circle) with an optional informal physical activity component such as a walk or urban poling session run by the Program Facilitator; (2) ongoing Program Facilitator contact to encourage healthy activities, address concerns as needed (e.g., providing community resources) and weekly step count recording; (3) continued encouragement and engagement via social media; (4) an end of program celebration with a local Elder.

During the nutrition and wellness education modules, “in-class” exercises are also provided (e.g., during Week 12 “Beverages” the Program Facilitator will bring in different beverages and granular sugar to demonstrate how much sugar is in each beverage). Moreover, dispersed through the nutrition education modules, with a specific focus in Week 5 “Honoring Culture and Traditions” attention is given to traditional foods (e.g., location-dependent wild game and berries). This focus on cultural nutrition remains present at celebrations throughout the program with local Indigenous caterers, Program Facilitators, and/or participants preparing local meals to share and connect.

Throughout the program, participants complete health education modules in the Nourish manual that support self-reflection, educate on nutrition and disease prevention, and support connection to local healthcare professionals. Again, because of the safety of the environment, the group can explore their barriers and facilitators to behavior change openly without stigma and identify specific needs and appropriate resources to address them with the goal being to increase activity and the ability to manage one’s own health. It is important to note that childhood trauma, addictions, and mental illness all greatly impact an individual’s ability to make positive behavior changes and through pre-program surveys and engagement with Program Facilitators, individuals with more severe, specific needs may be identified and appropriate referrals made. In each community, the Community Leads support the Program Facilitators to establish a comprehensive resource list of healthcare professionals.

## Evaluation

All measures for the program were approved by the advisory group. Ethics approval for this study was given by the Conjoint Health Research Ethics Board at the University of Calgary. All communities are asked if they have their own ethics processes in place, if so, ethics approval is sought from the local community. If the program is running on a First Nations reservation, then data collection and retention follows (OCAP)® principles of ownership, control, access and possession [20].

Primary and secondary objectives, as well as measurement periods, are summarized in Table 2.

**Table 2:** Outcome measurements over time

Timepoint	Measurements
Time 1 (3-month pre-program)	Participant/patient activation (PAM™ score and level) Physical activity (N-IPAQ, step counts) Well-being (mental health continuum—short form©) Fitness (grip strength, 1-mile walk test) Community walkability (PANES) Anthropometrics (weight, BMI, % body fat, waist circumference) Mental health and well-being (CHMS sense of belonging question GEN_Q18) Quality of life (EQ5D5L™) Cardiometabolic health (blood pressure, resting heart rate, hemoglobin A1C/fasting glucose, lipid panel) Medication change Smoking status CANRISK score Metabolic syndrome (MetS) Healthy eating index—short form Food security—2-item food security screening tool
Time 2 (Immediately pre-program)	Participant/patient activation (PAM™ score and level) Physical activity (N-IPAQ, step counts) Well-being (mental health continuum—short form©) Fitness (grip strength, 1-mile walk test) Anthropometrics (weight, BMI, % body fat, waist circumference) Mental health and well-being (CHMS sense of belonging question GEN_Q18) Quality of life (EQ5D5L™) Cardiometabolic health (blood pressure, resting heart rate) Medication change Smoking status Awareness or access to previously unused or unknown resources Healthy eating index—short form Consumption of traditional foods Nutrition knowledge Food security—2-item food security screening tool
Time 3 (Post live program)	Physical activity (N-IPAQ, step counts) Fitness (grip strength, 1-mile walk test) Anthropometrics (weight, BMI, % body fat, waist circumference) Mental health and well-being (CHMS sense of belonging question GEN_Q18) Quality of life (EQ5D5L™) Cardiometabolic health (blood pressure, resting heart rate) Food security—2-item food security screening tool
Time 4 (Post full program)	Participant/patient activation (PAM™ score and level) Physical activity (N-IPAQ, step counts) Well-being (mental health continuum—short form©) Achievement of balance (medicine wheel) Fitness (grip strength, 1-mile walk test) Community Walkability (PANES); anthropometrics (weight, BMI, % body fat, waist circumference) Mental health and well-being (CHMS sense of belonging question GEN_Q18) Quality of life (EQ5D5L™) Cardiometabolic health (blood pressure, resting heart rate, hemoglobin A1C/fasting glucose, lipid panel) Medication change Smoking status CANRISK score Metabolic syndrome (MetS) Awareness or access to previously unused or unknown resources Healthy Eating Index—short form Nutrition knowledge Food security—2-item food security screening tool Qualitative reflections using a photovoice
Time 5 (One year after full program)	Participant/patient activation (PAM™ score and level) Physical activity (N-IPAQ, step counts) Well-being (mental health continuum—short form©) Achievement of balance (medicine wheel) Fitness (grip strength, 1-mile walk test) Community walkability (PANES); anthropometrics (weight, BMI, % body fat, waist circumference) Mental health and well-being (CHMS sense of belonging question GEN_Q18) Quality of life (EQ5D5L™) Cardiometabolic health (blood pressure, resting heart rate, hemoglobin A1C/fasting glucose, lipid panel) Medication change Smoking status CANRISK score Metabolic syndrome (MetS) Awareness or access to previously unused or unknown resources Healthy Eating Index—short form Nutrition knowledge Food security—2-item food security screening tool Qualitative reflections using a photovoice

The effectiveness of the Makoyoh'sokoi program in improving objective measures of physical activity, health status and patient-reported outcomes will be assessed by comparing measurements made at multiple timepoints.

### Primary measures

Primary measures include participant activation (PAM™), objectively measured physical activity (pedometer step count), self-reported physical activity (The Neighbourhood-International Physical Activity Questionnaire [N-IPAQ]), participant mental health status (Mental Health Continuum-Short Form [MHC-SF©]) and overall balance based on Medicine Wheel philosophy.

The PAM™ score has been used to assess effectiveness of diabetes prevention programming in Indigenous populations [21]. It is a well-validated tool used to assess the effectiveness of interventions for many chronic diseases, and small increases in PAM™ score have been shown to correlate to reduced healthcare costs and improved health status. However, there is less information about it being applied to lay-programming, specifically to primary prevention and to Indigenous populations [21].

Daily step counting has been shown to be an enjoyable and pragmatic method for encouraging physical activity and correlates to mortality in the general population; specifically in women [22, 23]. Walking is generally inexpensive and readily accessible. We will take attendance and use pedometers to track weekly step counts. Pedometers were chosen to assess physical activity because they were successfully used in the pilot programs, provide immediate motivational feedback to participants, and are cost effective and validated. Of note, some of the communities have concerns regarding walkability and ways to address this will be explored in each community. For this reason, the N-IPAQ and Physical Activity Neighborhood Environment Survey (PANES) were included. N-IPAQ is a reliable, validated, and easy to administer questionnaire that measures neighborhood-based physical activity. It has been tested online, comparing walking scores among Canadians [24]. The PANES provides estimates of self-reported neighborhood, built environment supportiveness for physical activity with some degree of test–retest reliability and construct validity. A study was completed using paper and online versions of PANES in Canadian adults, and it was found to be reliable for capturing overall agreement for perceptions of the neighborhood-built environment [25].

The MHC-SF© has a strengths-based focus and uses three categories (languishing, moderate, flourishing). It has previously been used in Indigenous populations and found cultural activities were positively correlated to MHC-SF scoring. [26]

Qualitative Medicine Wheel exercises are to be completed monthly during the program. Participants will be asked to reflect on the question:

What aspects of your mental, spiritual, physical, or emotional health did you come to value through the program? And has becoming more physically active increased your awareness of other aspects of your health?

A peer-researcher will be identified during the second month of each program. The peer-researcher will be trained in the photovoice method. Peer-researchers will meet with participants and educate the participants on how to create a photovoice to answer the question.

### Secondary measures

Secondary measures include health-related fitness measures including cardiorespiratory, muscular, morphological, and metabolic fitness. Social well-being, Quality of Life, nutrition knowledge, and awareness and access to health supportive services will also be measured.

Cardiorespiratory fitness will be measured with the one-mile walk test. Muscular fitness will be measured with hand grip dynamometry. Morphological fitness will be measured using bioelectrical impedance (BIA), waist circumference (WC), and body mass index (BMI). Metabolic fitness will be measured through biochemical markers (hemoglobin A1C, fasting glucose, cholesterol [total, LDL-c, HDL, TGs]), blood pressure (BP) and heart rate (HR) measurements. Composite scores will be taken indicating presence or absence of metabolic syndrome as well as risk score calculations including the Canadian Diabetes Risk Questionnaire (CANRISK) score.

To evaluate social well-being, a question taken from the Canadian Health Measures Survey (CHMS) that measures a sense of belonging to local community will be used. Effect on quality of life will be assessed using the EQ-5D- 5L™.

Awareness and access to services (e.g., cultural programming, mental health, and medical supports) will be measured. Both nutrition knowledge and eating patterns will be assessed, the Healthy Eating Index Score (HEI), as well as the Short HEI will be used to assess the latter, traditional food frequency and intake will also be assessed. The HEI has been adapted to a Canadian context [27]. The higher the overall HEI score, the better the diet quality. The Short HEI has been systematically developed and validated to provide a brief tool that assesses diet quality [28].

Noting the current mixed methods evaluation and the elements described in our earlier work, applying realist theory, can inform both outcomes and mechanisms. We will also track adaptations made by each unique program in order to assess ecological validity and cultural sensitivity, using a framework adapted from Bernal et al. [29]. Viewing programing from a community specific and realist lens can inform future program development. For example, collective effort helped support self-actualization through accountability, health knowledge translation, and culturally appropriate transfers of knowledge [19].

## Conclusion

Physical activity is an important health promotion tool for combatting chronic diseases. Canada’s Truth and Reconciliation Commission emphasizes reducing barriers to participation in physical activity and recommends the creation of culturally relevant and supportive policies and programming. Makoyoh’sokoi is a holistic wellness program, developed and led by Indigenous women to support their health and well-being. The implementation of health and wellness programs that are grounded in Indigenous ways is paramount to fulfilling commitments laid out in the TRC and improving the health and well-being of Indigenous women.

## Limitations

The main limitation of our program is that our cultural engagement was restricted to Canadian Indigenous populations, and specifically those located in Alberta and Saskatchewan. It is recommended that communities that want to implement similar programing consult their regional and local Indigenous communities to help modify the program to fit their needs.

## Data Availability

All data generated or analyzed during this study are included in this published article.
